# The final frontier: what is distinctive about the bioethics of space missions? The cases of human enhancement and human reproduction

**DOI:** 10.1007/s40592-022-00164-6

**Published:** 2022-10-28

**Authors:** Konrad Szocik, Michael J. Reiss

**Affiliations:** 1 Interdisciplinary Center for Bioethics, Institution for Social and Policy Studies, Yale University, Prospect St. 238, New Haven, CT 06511 USA; 2 Department of Social Sciences, University of Information Technology and Management in Rzeszow, Sucharskiego 2 St., 35-225, Rzeszow, Poland; 3 University College London Institute of Education, London, England

**Keywords:** Space bioethics, Military ethics, Autonomy, Human enhancement, Reproductive ethics, Rights, Mars, Space philosophy

## Abstract

We examine the bioethical issues that arise from long-duration space missions, asking what there is that is distinctive about such issues. We pay particular attention to the possibility that such space missions, certainly if they lead to self-sustaining space settlements, may require human enhancement, and examine the significance of reproduction in space for bioethics. We conclude that while space bioethics raises important issues to do with human survival and reproduction in very hazardous environments, it raises no issues that are distinct from those in terrestrial bioethics. Rather, space bioethics raises extreme versions of bioethical issues that are already found in the military, when working in extreme environments (such as Antarctica), or when living in circumstances (such as in prison) where one’s autonomy is severely curtailed.

## Introduction

In a world where material accumulation has often seemed an unquestioned sign of progress, it is not surprising that there has more recently been something of a rejection in popular culture of this philosophy (Marie Kondo, the decluttering movement, etc.), with a move more towards the enjoyment of experiences than the enjoyment of object possession. One set of experiences that has exploded in popularity (at least in pre-COVID-19 days) for people of all ages—from pre-university backpackers to those on post-retirement cruises—is travel experiences to and in distant places. In this paper we envisage space missions as an extreme (perhaps the ultimate) type of travel and explore (an appropriate word for travel in general and space missions in particular) the bioethical issues associated with such travel.

There is a growing literature on the ethics of travel. Much of it, while important, is conceptually straightforward and is principally to do with reducing damage to the environment. In a more nuanced analysis, Islam saw travel as a mode of encounter with difference but noted that it often isn’t this, as the traveler, despite having moved in space [Islam’s words], doesn’t seem to have moved at all (Islam 1996). To give concrete expression to this point, we can note how much of the food served in and near the port of Calais in northern France is British, catering to the hordes of tourists who arrive from England. More recent discussions on the ethics of travel examine it through a range of lenses including colonialism and literary theory (Fowler et al. 2014).

Human space travel raises ethical issues, including bioethical ones. In this paper, we have in mind long-term and deep-space missions which may include colonizing planets or other extraterrestrial bodies such as moons. Perhaps the most interesting ethical issues philosophically, but also the most challenging politically, will be ones related to human biology and psychology. This is why we concentrate on space bioethics rather than the more general field of space ethics (which includes social and political philosophy and environmental ethics). We are also interested in attempting to determine whether space bioethics really demonstrates novel problems in bioethics. Are bioethical issues in space qualitatively different from bioethical issues on Earth? Alternatively, is it rather that the differences—if any—lie only in different, specific environments, in frequency, or in biases towards different ethical norms in similar situations on Earth?

If this latter possibility (i.e., that space bioethics is not fundamentally distinct from Earth bioethics) proves to be the case, space bioethics can be thought of as being analogous to military bioethics. Military bioethics is a domain of bioethics that applies to a narrow section of the population, focused on specific issues but issues that still arise more generally in non-military contexts, albeit under different local conditions and/or with different frequency. (For example, issues to do with killing, and whether to obey commands that one believes to be bad arise in civilian life as well as in the military.) Even if there is nothing fundamentally distinctive about space bioethics, the label ‘space bioethics’ is likely still to be useful because it focuses attention on a specific part of human activity where some bioethical issues happen more often and/or are solved differently than on Earth—even if they remain the same at a fundamental level.

## Bioethical issues in space

We start by sketching possible bioethical issues which may arise in future space missions. We use the word ‘may’ because while we now have 60 years of knowledge about bioethical issues in space—since Yuri Gagarin on 12 April 1961 became the first human in space, making his 108-minute orbital flight in Vostok 1—it remains the case that thought experiments and science fiction are important resources, given that actual knowledge of humans in space is still somewhat modest (including the NASA flights to the Earth’s moon and back and the dozen or space stations that we have had to date, beginning with Salyut 1). Both science fiction (since the days of Mary Shelley’s *Frankenstein*, published in 1818) and thought experiments (e.g., Judith Thomson’s (1971) violinist and Derek Parfit’s (1984) repugnant conclusion) have a long history of being used in bioethics.

We build on our arguments in previous writing where we have explored the extent to which space travel may provide reasons in support of human enhancement and discussed whether a feminist framing for space bioethics is especially appropriate (Norman and Reiss 2020; Szocik et al. 2020; Szocik 2021). We examine here two areas within which there might be fundamentally new bioethical issues raised by space missions: human genetic enhancement and reproduction in space. We note that one of these, human genetic enhancement, is likely (for the foreseeable future) to be undertaken pre-launch (the technologies for human genetic enhancement to be undertaken on a space ship or at a space colony do not at present exist) and may take place in the near future, given the increasing amount of work, which we discuss below, presently taking place on human gene therapy. The other area, reproduction in space, while manifestly not necessarily requiring any new technologies, is likely, on the grounds of safety, to be a longer-term possibility. Finally, we acknowledge that all this talk of space missions and the attendant bioethical issues may sound far-fetched. Nevertheless, aside from the inherent intellectual interest that these issues raise, they may come to pass under certain conditions and there is much to be said for examining the bioethical implications of issues before such issues actually arise.

Of course, space missions generate many more significant bioethical challenges. One of them is the issue of genetic screening, which may be mandatory and find specific justification in the difficulties of the mission. But such a requirement, if it were to be applied to broader groups for far more extensive space colonization, could interfere with the right to open space exploration and equality. The idea of open and equal access to space would also be threatened by what are likely to be other requirements, such as undergoing mandatory pre-flight medical checks. Finally, the inevitable limitations on the medical resources available during space missions raises issues about selection criteria for treatment, the resolutions of which are likely, we suspect, to be utilitarian in nature. While all of these issues are important, the subject of our paper is the human enhancement and human reproduction issues likely to arise on long-term space missions (think the Earth’s Moon and especially Mars).

### Human enhancement

Let us imagine that some humans conclude that there is a huge incentive to go to space. Such incentives may include economic benefits from space mining and space tourism, political benefits associated with military dominance in space, or, in the long-run, establishing one or more permanent space settlements that work as back-ups for the continued existence of the human species in the event of a disaster on Earth that eliminates humankind (such as a nuclear winter or a pandemic that really does make COVID-19 look like a mild case of influenza). All such incentives are strong even if their moral status differs substantially. Nonetheless, a strong enough motivation to realize long-term human space missions may be deemed to justify certain reproductive policies on Earth. People may want to design (this is the more controversial and invasive option) or at least identify and select the best candidates for such missions.

As humans become an interplanetary^1^ species, or at least a species that travels often and with relative ease in space, a reproduction policy on Earth that is based on more than simply the identification and selection of suitable travelers may be deemed an acceptable tool and, as such, become less controversial and fantastic than it may seem to be today. To see more clearly how important socially such reproductive policies as germline gene editing or embryo selection aimed at designing humans for space may become, it is worth referring to a thought experiment known as the ‘risk of human obsolescence.’ Robert Sparrow talks about a so-called ‘yesterday’s child.’ This is a hypothetical child in the future who—at the embryo stage—has been genetically modified at her parents’ behest so that she has one or more enhanced (upgraded) feature. However, a few years after she has been born, rapid progress in gene editing technology offers better pre-natal upgrades of the same feature (Sparrow 2019). It is assumed here that such gene editing is technologically feasible and legally available. Another assumption is that only germline gene editing can realistically offer the desired effect. Here, we do not discuss the ethical importance of such issues as inequalities in access to these new technologies. However, even without such inequalities in access (which might arise from economic considerations or because certain categories of parents refuse to avail themselves of these new ‘designer baby’ technologies for religious or other reasons), being excluded from benefits offered by more advanced versions of gene editing may be a problematic issue, assuming that these upgrades are not available somatically. In such a scenario, a future society will have cohorts of people with differently levels of capabilities; yesterday’s child is therefore a disadvantaged child.

Of course, this is only a thought experiment which does not take into account many other factors such as the actual efficacy of germline gene editing for purposes other than therapy and disease prevention. Many factors that are traditionally discussed by philosophers as possible targets for human enhancement, such as intelligence, probably cannot, or can only to a small extent, be modified by genetic engineering (Reiss 2021). The contribution of any one gene locus is small or very small relative to the impact of environmental and other factors, including upbringing, education and chance. For these reasons, this thought experiment of yesterday’s child is far from perfect but it draws attention to issues of equity that will arise should substantial human enhancement that goes beyond therapeutic considerations ever be implemented.

Having in mind this ‘risk of human obsolescence,’ it is worth examining issues to do with human enhancement in relation to human space missions. There are good reasons to assume that this is an issue which will affect the future adult lives of not-upgraded children to a far greater extent in space than on the Earth. In the context of space travel, a risk of genetic obsolescence is not an issue if an individual who has been genetically modified as an embryo then goes off into space (without gene editing in space being a possibility). While we can imagine a scenario where progress in space medicine and genetics will offer every couple of years new methods of germline gene editing, an issue arises if such substantial embryo modifications are realized. Such modifications for the purposes of space missions are likely to be grounded in medical reasons and will be treated as a countermeasure to hazardous factors in space. Two of these hazardous factors are non-Earth gravity and space radiation. We can imagine a scenario in which gene editing to enhance individuals’ abilities to tolerate these factors is considered necessary for the success of space travel, and thereby becomes mandatory.

However, mandatory gene editing (whether germline or somatic) introduces different kinds of ethical issues than does optional gene editing. Here, we consider the scenario of optional gene editing for space travel. Such genetic modification may be recommended due to expected risks in space but, as long as it is not mandatory, future space travelers have a right to travel in space with or without gene editing. Other hazardous factors in space such as distance from Earth, habitat confinement or isolation, while important for mission success, may not be targeted by space genetics. Let us therefore assume that deep-space missions will be possible for non-genetically modified recruits. However, their genetically modified colleagues will be better equipped for such space missions, even down to possibility simply of surviving. We can see that the context of space missions affords a qualitatively different scenario than do apparently parallel contexts on Earth. On Earth, we are talking about genetic obsolescence leading to relative disadvantage (for instance, with regards to intelligence); in space, we may be talking about genetic modification substantially enhancing the chances of remaining alive.

Let us, for the sake of argument, imagine that humans will have realized one or more well-developed programs of interplanetary missions 50 years from now, i.e., in about 2075. Let us further imagine that germline gene editing that targets health issues in space (e.g., tolerance of non-Earth gravity and high levels of radiation) becomes available from about 2040 and that astronauts are almost never younger than their mid-twenties. Children born in the late 2040s who have received germline modification that successfully targets one or more of these various health issues in space are greatly advantaged relative to other potential astronauts. Other children born at about the same time or earlier who also want to go into space, but who have not been modified for whatever reasons (economic, ideological, the technology was not available, etc.), are likely to be disadvantaged as potential astronauts, just as individuals with sickle cell anemia are nowadays (in the early 2020s) disadvantaged if they want to become air pilots. The issue in 2075 is not the risk of being obsolete when compared with children born in 2060 (let’s assume that some, at least minimal, improvement in space genetics will be possible), because children born in 2060 are too young to be chosen as astronauts in 2075. The border is clear and simple: some potential future astronauts and space settlers will possess genes better adapted to space radiation and non-Earth gravity, while others will never possess such genes. For the sake of argument, we have assumed that alternatives to germline gene editing to enhance tolerance to the health hazards of space are not available, or are substantially less effective in some regard. Cost effectiveness may also be an issue here because germline gene editing may be substantially cheaper than alternatives such as faster space ships—which could greatly reduce the time taken for space travel—or ‘artificial’ gravity.

We can imagine a scenario where not being genetically modified as an embryo (or in some other way enhanced) excludes a person from any space mission (or certain types of space missions—e.g., ones beyond the inner reaches of our solar system) as an adult. It is therefore possible that human enhancement may be a requirement for such space travel. We do not discuss here the important issue of fair and equal access to space which, according to some authors, should not exclude anyone only because they are not genetically modified (Schwartz 2020). If germline gene editing is the only effective way of adapting humans to long-distance space missions, it is easy for some individuals, by virtue of their social circumstances, to lose the opportunity for space travel when living in a spacefaring country where germline gene editing is practiced.

**Table:** 

Similarities and differences in bioethical issues on Earth and during space missions using human enhancement as an example	
Similarities	Differences
Limitations on autonomy in space missions are similar to those in the army, prison and Arctic expeditions	Human enhancement on Earth is more often considered for non-health purposes than in the context of space exploration
Narrowly targeted space missions are like military missions, where specially trained and selected personnel may be subject to special requirements, and their rights and responsibilities are governed by separate codes of ethics	Human enhancement in space may be mandatory
Human enhancement applied for the purposes of space missions is analogous to the various types of enhancement applied to soldiers for the purpose of increasing their performance and endurance geared toward mission targets	The risk of ‘obsolete’ enhancement is greater in a space mission society than for terrestrial situations

### Reproduction

The demands of human reproduction, both pursuing childbirth and preventing it, are disproportionately impactful on women, relative to men. Reproduction in space raises some distinctive health risks, and may additionally result in women being coerced to a greater extent than would be usual on Earth. For example, in terms of coercion, there may be pressure brought to bear, even a requirement, for women to have an abortion if they become pregnant on a long space flight. At the other extreme, there may be pressure bought to bear on women in a space colony to produce children. In terms of reproductive ethics, regulating the choices of individuals to reproduce or not reproduce lies in tension with well-established positions on the importance of reproductive autonomy, either as a component of broader appeals to life projects or as a matter of rights.

At the same time, considerable constraints on reproductive autonomy have occurred in certain situations on Earth. China’s so-called ‘one-child policy’ ran from 1979 to 2015 (though during 30 of those years about half of Chinese couples were permitted to have two children) and is a famous example. At the other extreme, a number of countries have tried to persuade couples to have large numbers of children, with both ‘carrots’ (financial incentives) and ‘sticks’ (including restrictions on contraception and abortion—as happened with Decree 770 in Ceaușescu’s Romania in 1966). In most countries, prisoners (whatever their gender) are not allowed the opportunity to try to reproduce.

Some parallels to the ethics of human reproduction and reproductive autonomy in space can also be found in the context of military service on Earth. Such issues as reproductive autonomy for individuals in the military, their rights to services including family planning, and how their professional obligations may impact their reproductive choices, may be used as an analogy to space missions. In the military of many countries, individuals who wish to become pregnant have the choice to do so (and to continue or not with their pregnancy), but it is often held to be important that medical services provide access to routine and comprehensive family planning services so that those same individuals can exercise their responsible choices in the light of their professional obligations. When one takes into account constraints typical for a military service such as pregnancy policy on deployment (Ritchie 2001), it seems clear that the space environment does not necessarily present a fundamentally novel challenge. This is also the case if one considers expeditions on Earth to places where medical support from outside is difficult or even impossible at certain times of the year (an on-going issue for over-wintering scientists on Antarctica).

There is little doubt that the risks of pregnancy are higher in space than virtually anywhere on Earth due to environmental factors. There are therefore good reasons to suppose that hazardous factors in space may create a new ethical situation, distinct from anything known on Earth. In contrast to hazardous environments on Earth which may be risky for pregnancy (like military service, Antarctica, prisons, abusive relationships, special requirements of particular jobs, etc.), space environment is hazardous permanently and irreversibly. It is simply impossible to change that environment, or to take parental leave and then come back to work after pregnancy. This is a feature which creates a particular bioethical ‘scenery’ in space. We presume that once a future female astronaut and/or settler of child-bearing age decides to participate in a space mission, her reproductive autonomy to choose to reproduce or to choose not to reproduce may be substantially reduced.

Let us assume, for the sake of argument, that human reproduction in space will be possible only for enhanced humans, on the grounds that standard human reproductive biology is too sensitive to work correctly in space, given high doses of space radiation and non-Earth gravity. For reproductive autonomy in space, it does not much matter if such ‘enhancement’ is perceived more as therapy or as enhancement, traditionally understood. It also does not much matter if human reproductive rights are understood as basic (i.e., inalienable), or as having a certain element of conditionality (e.g., as voting rights are—as these depend on a person’s age and, in some countries, are not granted to prisoners and even certain categories of ex-prisoners). What matters is simply that human reproduction in space will require special modifications. These modifications (enhancements) might apply to men as well as to women [given our increasing understanding of the sensitivity of sperm to environmental conditions (Almeling 2020)], though it may be premature to presume that such modifications will necessarily be irreversible, given the rapid rate at which human reproductive technologies and genetic interventions are advancing.

The possibility of genetic obsolescence, introduced above, may be of particular relevance for reproductive issues in space. Let’s assume that germline gene editing on Earth, at the pre-launch stage, will be needed to enable subsequent human reproduction in space when such a genetically modified embryo eventually becomes an adult, whether still on a mission or in a colony. In such a scenario, radical human modification of germline cells may lead to a specific kind of exclusion in adult life, not to social exclusion or exclusion in the labor market (which may happen in a yesterday’s child scenario on Earth), but to a reproductive exclusion. Such a reproductive exclusion would apply not to the individual(s) who have been genetically modified but to those—e.g., older crew members—who have not. Reproductive exclusion on a (very long) space mission or in a space colony—if the possibility ever existed due to there being a mixture of humans, some suitably enhanced for long-term existence in space, others not—will be a specific kind of exclusion distinct from that on Earth. On Earth, different kinds of exclusion caused by a genetic ‘enhanced rat race’ (Sparrow 2015) can lead to an on-going situation of exclusion of the current generation (of both enhanced and not-enhanced individuals) by each and every succeeding generation of enhanced individuals (assuming that such upgrades will continue to be possible).

Such exclusion, however, will not necessarily lead to a reproductive exclusion. It is worth keeping in mind at least two possible meanings of the term ‘reproductive exclusion.’ First, reproductive exclusion (such as that which may happen on Earth) may only be social reproductive exclusion. Such exclusion may happen when a not-enhanced individual, or an enhanced but obsolete individual, will not be as attractive as a sexual partner. (S)he will be lost on the mating market—just as happens nowadays, especially in countries with skewed adult sex ratios or where polygamy is widely practiced. Secondly, reproductive exclusion (such as that which may happen in space) may be a biological kind of exclusion. Individuals who are not modified genetically may be excluded from the very possibility of safe reproduction in space. (This, of course, does not mean that they would be forbidden from engaging in sexual intercourse; rather, there might be a requirement for such individuals to use effective contraception—as happens nowadays in a range of situations, some considered ethically appropriate, others not.)

To some extent, reproductive constraints will be dependent on available technology and mission design. Reproductive constraints in space are environmental constraints and, theoretically, they could be overcome by technology. Such technological countermeasures might include thick shielding walls (much thicker than currently applied in space missions), artificial gravity or invasive methods of human enhancement, possibly including germline gene editing. It is possible that countermeasures which are available today, or in the near future, will provide relative safety for the crew of a mission to Mars (one of our nearest planetary neighbors and the one most likely to host humanity’s first extraterrestrial space colony). However, existing countermeasures may be ineffective to enable human reproduction, either en route to Mars or on Mars. This does not matter if Mars is seen as akin to today’s International Space Station or one of its predecessors, where a human presence has been maintained for a number of years without the need for human reproduction in situ.

If, though, a colony on Mars was established as or became a space refuge (Szocik et al. 2020), helping to ensure the long-term survival of humanity as a species, then there would indeed be a need for human reproduction in situ. Humans may live as a multi-planetary species with the overwhelming majority of humans living on Earth, as long as no catastrophic scenario happens on Earth, including economic collapse. Economic collapse may make any transportation system between Earth and its space colonies (or colony) economically infeasible or technologically impossible. Due to possible collapse on Earth (environmental, military, or economic), which may happen suddenly and unexpectedly, a space colony might change its status from something like a research station or space mining settlement to a (or the only) refuge of humanity. Political expediency can affect the implementation of conclusions reached through bioethical reasoning. We could imagine a scenario in which reproductive autonomy is considered as a basic human right, with long-term space settlements that do not respect such autonomy being prohibited; similarly, long-term space settlements that require substantial human enhancement such as germline gene editing, could also be banned. However, it seems entirely possible that warning of an imminent collapse on Earth might cause a rapid reversal of such bans if one or more long-term space settlements already existed and were therefore seen as the future of humanity.

It is clear that space bioethics, while in its early days, is emerging as a very context-dependent discipline. While one could argue that bioethics is always context-dependent, space bioethics seems to be especially strongly rooted in technological capabilities. The final point we would make in this section is that it appears that space bioethics, compared, for example, to conventional medical bioethics, may give more weight to utilitarian considerations and less to such deontological considerations as informed consent and autonomy. We have already seen that space bioethics may be prepared to contemplate germline alterations (to which, of course, while the participating individuals can consent, their offspring and subsequent generations cannot). This does not mean that space bioethics does not assign inherent moral status to individual human beings, rather that the extreme circumstances of space travel and existence may give particular weight to considerations above the level of individuals, such as the value of human survival as a species. This does not mean that autonomy within space bioethics is seen as valueless but that autonomy in space bioethics may be trumped by other values. Returning to the question we raised at the beginning of the paper about whether space bioethics really demonstrates novel problems in bioethics, the only one that seems truly novel relates to the possibility of human extinction, though even here such a possibility has been taken seriously by ethicists ever since the advent of nuclear warfare (Rotblat and Ikeda 2006).

**Table:** 

Similarities and differences in bioethical issues on Earth and during space missions using human reproduction as an example	
Similarities	Differences
The likely restrictions on reproductive rights in a future space colony are comparable to those known from history (including the one-child policy, eugenics, and depriving prisoners of their right to reproduce)	The constant presence of hazardous environmental factors in space provides a special context that can justify a radical permanent restriction of reproductive rights for all in a space colony (unlike restrictions on Earth, which were and are more likely to be time-limited and applied to specific groups)
The possibility of third parties influencing the reproductive decisions of prospective parents in a space colony is like the eugenics policies of the first half of the 20th century in Western countries	Gene editing of prospective parents may be an essential and necessary condition for successful reproduction
The application of germline gene editing during space missions is likely to be controversial, as is the application of germline gene editing on Earth	Germline gene editing of children born in space may be mandatory for environmental reasons
The idea of subordinating the reproduction of individuals for the good of the group in space is like the idea of regulating the reproduction of individuals for the good of the population on Earth	Potential constraints on reproductive rights in a colony in space may be unacceptable on Earth

## Rationale for space missions as a significant factor in space bioethics

An important factor in space bioethics, along with the current state of technological capability, is the rationale for human space missions. Here, we consider only missions to Mars and beyond. Missions to the (Earth’s) Moon, while interesting for political and social reasons, are not such as interesting for bioethics as missions to Mars and beyond. The main reason lies in a fact that, at an astronomical scale, the Moon is very close to Earth. As such, relatively urgent evacuation will be available (barring human technological collapse on Earth) for astronauts in need [analogous to being rescued from Antarctica in mid-winter; cf. the Apollo 13 malfunction and compare the films *Apollo 13* (1995), *Gravity* (2013) and *The Martian* (2015)]. Human enhancement would be not necessary even for a permanent Moon base because a feasible alternative would be a shift work system with crew being replaced on a regular basis, as has happened and continues to happen on the various space stations [again, for film afficionados, see *Moon* (2009)]. Teleoperations and telemedicine are available in near real time whereas they are not possible between Earth and Mars, due to the communication delay between Earth and Mars which ranges from four to 24 min, depending on the relative positions of the two planets. Another important issue is the fact that travel to the Moon from Earth only takes about 3 days. As such, space travelers are not exposed to the impact of cosmic rays and reduced gravity for the minimum of 6 months that it currently takes to get from the Earth to Mars or vice versa. Last, but perhaps not least, some social and psychological stressors such as isolation and distance from Earth will be less challenging on our Moon than on Mars. We now examine the implications, if any, that missions to Mars and beyond have for the moral norms of human autonomy, reproductive rights, and human enhancement.

### Limits to human autonomy

A common-sense assumption in space bioethics would suggest that limits to autonomy are inversely proportional to length of mission. Military ethics once again provides an analogy. Soldiers are prone to (expected to) self-sacrifice during their missions and, as such, accept substantial restrictions on their autonomy (to the extent that it is common for soldiers to be subject to court-martial on the rare instances that they overtly disobey orders). However, the longer a space mission, the less likely it seems that astronauts and space settlers will accept substantial constraints on their autonomy, or even be expected to. It is not only military ethics that offers opportunities to explore constraints on autonomy; however, in a non-military context, such constraints are likely to be even more temporary and require explicit and strong rationales. Consider, for instance, restrictions in policy that addressed the early stages of the COVID-19 pandemic. Many governments imposed lockdown policies but strict lockdowns usually lasted for only a relatively short time, typically a matter of a handful of weeks, or a few months. Such restrictions on autonomy are accepted (by most people) as the consequence of a necessary tradeoff between freedom and safety (Mill 1859). What, though, is important to note is that these autonomy constraints are (mostly) accepted in large measure because they are intended to be temporary; democratic societies do not rapidly transition into totalitarian states (we hope).

Space as an environment is less flexible than is Earth in relation to minimizing autonomy constraints. For a start, dependence on life support systems and limited resources cause one kind of autonomy constraint (one cannot simply choose on a whim to ‘pop out for a bit of exercise’). However, another kind of autonomy constraint, again rooted in environmental factors in space, will serve to remove basic human choices, including those to do with reproduction, which humans on Earth usually evaluate as inherently connected with being human.

One of the ethical issues considered within military ethics is that of the duty to obey orders. Insofar as orders must be obeyed, and on this there is agreement in principle—that is, it is that scope of action to which society as well as the soldiers themselves voluntarily agree that they (the soldiers) are giving up some of their autonomy—then under certain conditions, a soldier has an ethical right not to obey an order. Such an instance is when the execution of an order leads to unnecessary harm or unnecessary death (the mere risk of suffering harm or death is accepted within military ethics if it is deemed necessary) (Visser 2003, 262–263). How would such a situation look in the case of space missions? Paradoxically, this exception, if transferred directly from military ethics to space mission bioethics, should justify even the mandatory application of radical human enhancement, assuming it is medically justified. That is, an astronaut has no right to refuse an assignment or to participate in a mission at all if she has been genetically modified. Moreover, the execution of the overriding order, that is, participation in the mission, here presupposes consent to one of the conditions placed on astronauts, that is, human enhancement. Furthermore, an astronaut would have the right to resign from the mission and refuse to obey orders—we assume here a militarized structure of space missions and, for simplicity, we abstract from the consideration of scenarios assuming some degree of voluntariness and freedom, which will certainly also depend on the purpose of the mission (scientific, commercial, or aimed at protecting humanity)—in a situation where she would otherwise know about the dangers of a given mission and the failure of its organizers to apply the countermeasures that are available. In this case, such a countermeasure might be genetic modification, which nevertheless would not be applied by the mission organizers, for example, because of their conservative moral beliefs. This case is also complicated by the fact that the purpose of the mission may affect its moral judgment and may determine the astronauts’ possession—or lack thereof—of the right to refuse to obey an order. If an astronaut considers a science mission to be not worth sacrificing her life for, could she refuse to follow orders, or abandon participation in such a mission altogether? And perhaps an astronaut would have a moral right to refuse to participate in a mission or to carry out a particular dangerous order only if all protective measures, in this case radical human enhancement, were not applied, and therefore his eventual death would be necessary. However unresolved the question remains, the issue is whether a given type of mission in itself is worth the sacrifice of human life; what can be resolved here is only that under certain conditions the death of an astronaut may be necessary for the completion of a given task or the accomplishment of an entire mission even if all protective measures, including radical, morally controversial human enhancement, would have been applied by the mission organizers.

### Constraints on reproductive rights

Human reproductive rights in a space settlement will be constrained, given that human space missions will be realized with the minimum available technology needed to colonize space. In this scenario we assume that humans will go to space as soon as possible (though Joe Biden’s presidency may not pursue ex-President Trump’s ambition of a crewed mission to Mars in the 2030s). An alternative scenario is postponement of these missions to such point in the future when progress in technology will eliminate all, or at least the most challenging, constraints on safe human reproduction. Such postponement may not be likely due to economic, political, or existential pressures.

Constraints on reproductive rights in space seem likely to be of two kinds. The first and basic kind is, as discussed above, to do with biological constraints. Human reproduction in a space colony may be hazardous due to threats to the child, and possibly the mother, resulting from radiation damage and continuous exposure to low gravities (even on Mars, the gravitational force is only 38% of its counterpart on Earth). These biological constraints may well lead to human enhancement for reproductive reasons being considered a requirement. The second kind of constraint is associated with social and reproductive policy. Such possible constraints on human reproductive rights may include: prohibition of reproduction for some individuals, groups or the entire space population (depending on the availability of food and other resources); constraints on partner selection (if a space government deems it necessary to determine that certain features are desirable for future citizens); and very liberal or very conservative abortion and contraceptive policies (dependent on current and desired fertility rates and population sizes). This second class of constraints on reproductive rights may seem draconian, even unimaginable to some, but they are not very different to those that many individuals have experienced over the course of history, including those that obtained (and still do obtain) under conditions of slavery or other forms of severe coercion.

### Mandatory radical human enhancement

Mandatory radical human enhancement becomes an ethical issue even if human enhancement as such is desirable and widely practiced. By radical, we mean an enhancement that is invasive, irreversible, and possibly heritable (so we can imagine that certain sorts of somatic gene editing and brain implants are invasive and might be, to all intents and purposes, irreversible, so that they can be considered radical, but are not heritable, whereas germline gene editing is heritable). Non-radical means of human enhancement include such techniques as the use of performance-enhancing drugs. Voluntary human enhancement may give rise to ethical issues (certain types of cosmetic surgery, including breast enlargement, come to mind) but mandatory human enhancement is clearly more contentious. We now consider two circumstances (and these have some similarities) in which space missions might give rise to mandatory radical human enhancement.

#### Large-scale colonization

Let us imagine a scenario in which there is a mass migration of people (at least a few thousand—sufficient to minimize the risk of inbreeding depression) to a space settlement on Mars. Let us further suppose that this is not a lifestyle choice, unlike the several hundred thousand people who have retired to Spain from England, but a response to severe problems on Earth, and that the settlement becomes self-sustaining, so that immigration of people or goods from Earth is no longer required. The question arises as to whether such large-scale colonization is dependent on radical human enhancement.

It is possible that technologies of the future will make human enhancement unnecessary because other countermeasures will be sufficient for large-scale colonization. However, at present it seems likely that radical human enhancement would be needed—people are already considering whether humans can be genetically engineered to have the greater resistance to cosmic radiation that is found in some other species (Norman and Reiss 2020). If such radical human enhancement is a prerequisite for space travel, then it becomes mandatory in one of two senses. The simpler is that, given the huge expense of taking someone to Mars, not to mention the opportunity costs of not taking someone else, it seems self-evident that certain enhancement will be required, just as certain vaccinations are nowadays required for certain jobs or for obtaining certain travel insurance. The other sense in which such enhancement might be mandatory is that from the perspective of someone given the possibility of moving to Mars, when the alternative is dying on Earth, their freedom of choice is severely constrained in the same way that anyone’s is faced with a life-or-death decision. It is possible that in the future such radical human enhancement will be considered as a routine procedure which is not so much an ethical issue but a matter of social or public policy, rather as vehicle insurance is required nowadays for those who own motor vehicles. Of course, it is possible that such enhancement will be mandatory only for some types of space missions, such as narrowly targeted missions. A ethical declarations is mandatory for publication in this journal. Please provide an alternative.Sorry but I do not understand what you mean, and what kind of ethical declaration you are asking about. Can you please give an example?

#### Narrowly targeted missions

Narrowly targeted missions might include scientific, economic, or military missions. Such missions will include only specially trained and well-selected crew members whose participation will be voluntary. Consequently, radical human enhancement, even if mandatory for the purpose of such missions, is likely to be accepted by all members of such missions and not seen as a major ethical issue (we are all used to meeting certain conditions for employment—think of the requirement not to take performance-damaging drugs, such as alcohol, while undertaking a very wide range of jobs). Of course, one can imagine the scenario where some scientists or businesspeople assume that space science or business should be open to those who possesses appropriate credentials even if they are not prepared to undergo radical human enhancement. In such a situation it is possible that there could be exceptional circumstances that would lead to the requirement for radical human enhancement being waived. Consequently, narrowly targeted missions might not necessarily be free from bioethical issues. Indeed, it is more likely that such narrowly targeted missions will not entail astronauts or settlers permanently leaving Earth. In circumstances, where travelers return to Earth, radical human enhancement is not what is needed (given that our definition of ‘radical’ included the criterion of irreversibility).

## Conclusion

Space bioethics differs substantially from terrestrial bioethics. In particular, it raises issues to do with human survival and reproduction in very hazardous environments. Nevertheless, we conclude that it raises no issues that are unique. Rather, space bioethics raises extreme versions of bioethical issues that are already found in the military, when working in extreme environments (such as Antarctica) or when living in circumstances (such as in prison) where one’s autonomy is severely curtailed. At the same time, the real possibility that space missions may be needed to ensure the survival of humanity gives rise to bioethical issues which, even if not unique, are perhaps of greater importance than have previously been considered, with the possible exception of the bioethical issues raised by terrestrial nuclear conflict.

## Footnotes

1 We use this term without necessarily implying the only planets will be the destinations of these space missions.
